# Convalescent plasma therapy in COVID-19 critically ill patients during advanced phases of clinical trials and their preliminary results

**DOI:** 10.31744/einstein_journal/2021RW6186

**Published:** 2021-04-09

**Authors:** Fernando Anselmo de Oliveira, Mariana Penteado Nucci, Gabriel Nery de Albuquerque Rego, Arielly da Hora Alves, Luciana Cavalheiro Marti, Leopoldo Penteado Nucci, Javier Bustamante Mamani, Lionel Fernel Gamarra

**Affiliations:** 1 Hospital Israelita Albert Einstein São PauloSP Brazil Hospital Israelita Albert Einstein, São Paulo, SP, Brazil.; 2 Universidade de São Paulo Faculdade de Medicina Hospital das Clínicas São PauloSP Brazil Hospital das Clínicas, Faculdade de Medicina, Universidade de São Paulo, São Paulo, SP, Brazil.; 3 Centro Universitário do Planalto Central Apparecido dos Santos GamaDF Brazil Centro Universitário do Planalto Central Apparecido dos Santos, Gama, DF, Brazil.

**Keywords:** COVID-19, Coronavirus infections, SARS-CoV-2, Betacoronavirus, Immunization, Immunization, passive, Plasma, Convalescent plasma

## Abstract

The objective of this study was to highlight the global scientific effort to fight the SARS-CoV-2, addressing the preliminary results of passive immunization through convalescent plasma. We performed a search at the major databases of interventional clinical trial protocols about the transfusion of convalescent plasma in patients with COVID-19, as well as, published articles (n≥25), using the following search strategy: [(COVID-19 OR SARS-CoV-2 OR nCoV-2019) AND (Convalescent plasma OR Plasma exchange) AND (Treatment OR Therapy)]. A total of 24 interventional clinical trial protocols (advanced in phases II-III, III, and IV) were included in this review, as well as three studies that had enough outcomes to evaluate the efficacy of convalescent plasma therapy for patients with COVID-19. All interventional clinical trial protocols applied approximately 500mL of convalescent plasma (from single or more donations) in hospitalized patients, mainly in patients with severe disease associated with standard therapy for COVID-19, and compared to placebo or standard therapy plus specific drugs. Most of interventional clinical trial protocols are multicenter, and the phase IV studies are recruiting at intercontinental centers of North America, Oceania, Europe, but most are recruiting center inside their own county. The three studies published reported similar approach of convalescent plasma intervention with decrease in length of stay, mortality, with less than 4% of adverse events, mainly for treating critical cases with life-threatening disease. All advanced clinical trials focused on convalescent plasma therapy in patients with COVID-19 hospitalized in severe conditions, and the preliminary results provide strong evidence for therapy for the COVID-19 patients.

## INTRODUCTION

At the end of 2019, the severe acute respiratory syndrome coronavirus 2 (SARS-CoV-2) emerged, resulting in the coronavirus disease 2019 (COVID-19), which caused an unprecedented health emergency and declared a pandemic by the World Health Organization (WHO) on March 11, 2020.^(^^1^^)^ Up to September 2020, this pandemic had affected approximately 28 million and killed roughly 920 thousand people worldwide.^(^^2^^)^ In Brazil, more than 5 million cases and more than 130 thousand deaths have occurred so far.^(^^3^^)^

New approaches to the development of immunity transfer were quickly implemented in preclinical studies, and currently interesting results of clinical trials are beginning to emerge, aiming to improve the symptoms of COVID-19, a heterogeneous disease caused by SARS-CoV-2.^(^^2^^)^ Simultaneously, different vaccines are being developed and tested for effective disease prevention.^(^^4^^)^

Before SARS-CoV-2, the use of convalescent plasma (CP) had been investigated, with positive outcomes, in outbreaks of other viral infections,^(^^5^^)^ such as the pandemic influenza A (H1N1), in 2009,^(^^6^^)^ avian influenza A (H5N1),^(^^7^^)^ among others. Furthermore, some studies have demonstrated that CP antibodies can limit virus proliferation during the infection and support viral clearance, which is favorable for fast recovery of the disease.^(^^8^^)^

A recent review on plasma therapy in COVID-19 patients reported low frequency of severe adverse event, and improvement in clinical symptoms in some participants after plasma therapy, but the authors judged the risk of reporting bias.^(^^9^^)^

The high or low titers of neutralizing antibodies against COVD-19 can be managed to reduce patient’s symptoms and mortality. There are 24 advanced clinical trials in phases II-III, III, and IV reported in several countries using CP to treat these patients, and answer this question.^(^^10^^–^^33^^)^ Although passive immunization has been used for over a century to treat infectious diseases, the recent results pose challenges to set the best time of plasma extraction and donor choice, in addition to the cost of this whole procedure. This lack of information empowers the movements of antivaccine and antiplasma groups.^(^^34^^)^

## OBJECTIVE

To highlight the global scientific effort in the fight against SARS-CoV-2, addressing passive immunization through convalescent plasma and their preliminary results.

## METHODS

A search was performed until 14 September 2020 at ClinicalTrials.gov (https://clinicaltrials.gov/), Chinese Clinical Trial Registry (http://www.chictr.org.cn/abouten.aspx) and EU Clinical Trials Register (https://www.clinicaltrialsregister.eu/) for interventional clinical trials on CP transfusion, in patients with COVID-19, using the following search strategy: [(COVID-19 OR SARS-CoV-2 OR nCoV-2019) AND (Convalescent Plasma OR Plasma Exchange) AND (Treatment OR Therapy)]. Then, the same strategy was used to search for studies in PubMed^®^ and Scopus databases about the efficacy of CP therapy to treat patients with COVID-19.

### Inclusion and exclusion criteria

This review included clinical trial protocols (CTP) phases III and IV that addressed the development of therapies based on CP to treat COVID-19 patients by passive immunization, and studies that showed the efficacy of CP therapy applied in more than 25 COVID-19 patients. The reasons for excluding studies were as follows: CTP for observational studies, CTP involving vaccines, and CTP canceled, or not approved, until the searching date in the databases.

### Study eligibility, data extraction, data collection, and risk of bias assessment

The study eligibility followed the Preferred. Reporting Items for Systematic Reviews and Meta-Analyses** (PRISMA) guidelines.^(^^35^^)^

### Data analysis

All results were described and presented using the percentage distribution for all variables analyzed in the tables.

## RESULTS

### Study selection

After applying the search strategies in the databases, 170 CTP were identified (153 protocols at ClinicalTrials.gov, 14 at Chinese Clinical Trial Registry, and three at the EU Clinical Trials Register). The search strategy used the PRISMA.^(^^35^^)^

Based on established inclusion and exclusion criteria, of 170 protocols identified, 146 clinical trials were excluded after screening (130 protocols were phases I and II and 16 were observational), remaining 24 protocols selected from these databases. In total, 24 CTP were included in the present work for passive immunization for COVID-19 through CP therapy.^(^^10^^–^^13^^,^^15^^–^^33^^)^

Of the selected studies published in the databases mentioned above, only three studies had enough data that allow statistical analysis of the outcomes, to evaluate the CP therapy efficacy due to the number of patients with COVID-19 (n≥25).^(^^36^^–^^38^^)^

### Overview of clinical trial protocols for passive immunization for COVID-19

**Figure 1:**
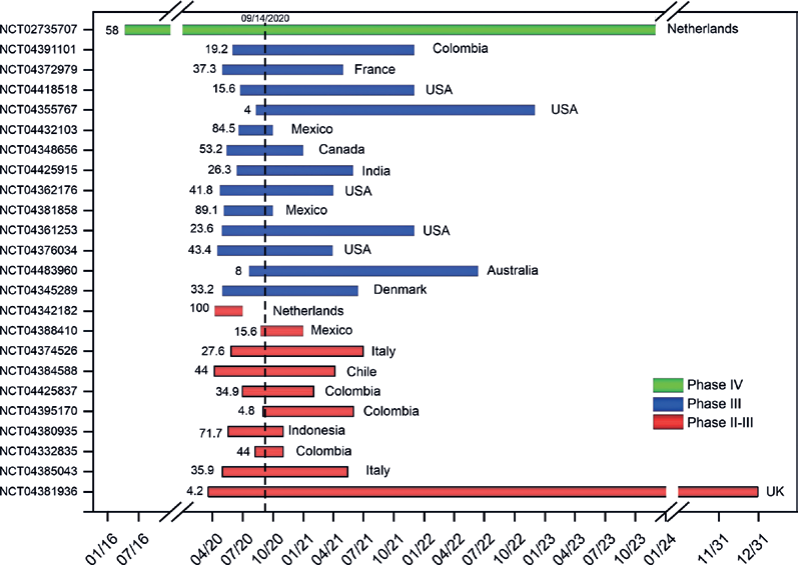
Analysis of the study progress rate percentile of clinical trial protocols on convalescent plasma therapy for hospitalized patients of COVID-19 distributed by their phases (green bar: phase IV; blue bars: phase III; and red bars: phase II-III) and the corresponding sponsor country

Of 24 more advanced clinical trials on CP therapy for COVID-19 inpatients, only one (4.2%) was in phase IV, with 58% of study progress rate (SPR), recruiting individuals in different countries by Netherlands sponsor (green bar of figure 1).^(^^10^^)^ The phase III studies (54.2%),^(^^11^^–^^13^^,^^15^^–^^19^^)^ almost half of CTP had more than 40% of SPR, mainly in Mexico^(^^15^^,^^19^^)^ and the United States (blue bars of figure 1),^(^^18^^,^^21^^)^ and the phase II-III studies (41.7%),^(^^24^^–^^33^^)^ one CTP has 100% of SPR in the Netherlands,^(^^24^^)^ and almost half of CTP have more than 40% of SPR (red bars of figure 1), as shown in figure 1 and table 1.

**Table 1:** Study design, arms, interventions, and study time progress

Identity number	Phase	Patient features	Intervention by arm	CP dose (day)	Age range (years)	Start date	Completion date	Progress (%)	Recruitment status
NCT02735707^(^^10^^)^	IV	Severe acute respiratory illness and severe community acquired pneumonia	Corticosteroid *versus* antibiotics *versus* macrolide *versus* Influenza antiviral *versus* LPV/Rit *versus* HydChl *versus* IFN-β1a *versus* Anak *versus* Tmab *versus* Smab *versus* Vit C *versus* heparin *versus* simvastatin *versus* CP *versus* ST	1 or 2 units within 48 hours	>18	4/11/16	12/1/23	58	Recruitingz
NCT04391101^(^^11^^)^	III	Severe patients treated in ICU	CP associated with ST *versus* ST	400-500mL	>18	6/1/20	12/1/21	19.2	Not yet recruiting
NCT04372979^(^^12^^)^	III	Early care of hospitalized patients	CP associated with ST *versus* SP associated with ST	200-230mL	18-80	5/1/20	5/1/21	37.3	Not yet recruiting
NCT04418518^(^^13^^)^	III	Early care for patients hospitalized	CP associated with ST *versus* ST	500mL of single-donor or 2 units of 250mL from 1-2 donations	18-70	6/24/20	12/1/21	15.6	Recruiting
NCT04355767^(^^14^^)^	III	Severe/critical hospitalized patients	CP (antibodies titers ≥1:160) *versus* placebo	CP: 1-unit *versus* placebo: saline with multivitamin	>18	8/11/20	12/1/22	4.0	Not yet recruiting
NCT04432103^(^^15^^)^	III	Severe/critical hospitalized patients	CP from IgG (severe patients *versus* critical patients) associated with ST	NR	>19	6/19/20	9/30/20	84.5	Not yet recruiting
NCT04348656^(^^16^^)^	III	Early care for patients hospitalized	CP *versus* ST	500mL of single-donor or 2 units of 250mL from 1-2 donations	>20	5/14/20	12/31/20	53.2	Recruiting
NCT04425915^(^^17^^)^	III	Severe/critical hospitalized patients	CP associated with ST *versus* ST	2 doses of 250mL on consecutive day started by day 3 of symptom onset	>21	6/14/20	5/30/21	26.3	Recruiting
NCT04362176^(^^18^^)^	III	Patients hospitalized or in ICU	Pathogen reduced CP *versus* placebo	CP: 500mL within 12 hours (day 0) *versus* placebo: 250mL of Lactate Ringers associated with multivitamins (day 1)	>22	4/24/20	4/1/21	41.8	Recruiting
NCT04381858^(^^19^^)^	III	Severe respiratory failure with invasive mechanical ventilation	CP (antibodies titers >1:164) *versus* HIg	CP: 400mL (2 units) *versus* HIg: 0.3g/kg/day (5 doses)	16-18	5/6/20	9/30/20	89.1	Recruiting
NCT04361253^(^^20^^)^	III	Patients hospitalized	CP (high-titer) *versus* SP (FFP or FP 24)	HT-CP: 2 doses of 250mL of single donor within 24 hours; FFP: 2 units of 200-275mL	>1	04/30/20	12/1/21	23.6	Recruiting
NCT04376034^(^^21^^)^	III	Mild, moderate and severe/critical severity	CP associated with ST *versus* ST	Adult: 200 to 250mL; children: 10mL/kg; 2 units severe patients or critical condition	>30 days	4/16/20	3/30/21	43.4	Recruiting
NCT04483960^(^^22^^)^	III	No severe patients	LPV/Rit *versus* HydChl *versus* CP	1 unit on day 1 and day 2	>18	7/21/20	6/12/22	8.0	Recruiting
NCT0434528 9^(^^23^^)^	III	Mild, moderate and severe/critical hospitalized patients	CP associated with ST (Smab *versus* baricitinib *versus* HydChl) *versus* ST associated with injective placebo	CP: (twice 300mL) and single dose of 600mL; placebo: (twice 300mL) and single dose of 600mL IV saline oral placebo: three times/day (7 days)	>18	5/1/20	6/15/21	33.2	Recruiting
NCT04342182^(^^24^^)^	II-III	Hospitalized patients	CP associated with ST *versus* ST	300mL (according to the Erasmus MC KIS protocol)	>18	4/8/20	7/1/20	100	Recruiting
NCT04388410^(^^25^^)^	II-III	Hospitalized patients with severe disease or at risk for severe disease.	CP *versus* placebo	CP: 2 units of 200mL within 24-72 hours); placebo: 200mL of saline	>18	8/25/20	12/31/20	15.6	Not yet recruiting
NCT04374526^(^^26^^)^	II-III	Hospitalized patients	CP associated with ST *versus* ST	200mL/day for 3 days	>65	5/27/20	6/30/21	27.6	Recruiting
NCT04384588^(^^27^^)^	II-III	Oncological and non-oncological patients with severe disease	CP associated with ST *versus* ST	1 or more units	>15	4/7/20	4/6/21	44.0	Recruiting
NCT04425837^(^^28^^)^	II-III	Hospitalized patients at high risk of severe disease or in ICU	CP (antibodies titers of ≥1:160) associated with ST *versus* ST	2 doses of 200mL in a day	>18	7/1/20	2/1/21	34.9	Not yet recruiting
NCT04395170^(^^29^^)^	II-III	Early care for patients hospitalized	CP associated with ST *versus* anti-COVID-19 HIg *versus* ST	CP: 200-250mL (day 1 and 3); 10% IgG solution: 50mL (patient ≥50kg); 1 mL/Kg (patient <50Kg), on days 1 and 3	>18	9/1/20	6/1/21	4.8	Not yet recruiting
NCT04380935^(^^30^^)^	II-III	Hospitalized patients in ICU (using mechanical ventilation)	CP associated with ST *versus* ST	NR	>18	5/18/20	10/31/20	71.7	Not yet recruiting
NCT04332835^(^^31^^)^	II-III	Moderate and severe/critical severity	CP associated with ST *versus* HyChl associated with ST	250mL on days 1 and 2	18-60	8/8/20	10/31/20	44.0	Not yet recruiting
NCT04385043^(^^32^^)^	II-III	Severe/critical hospitalized patients	CP associated with ST *versus* ST	NR	18-60	5/1/20	5/15/21	35.9	Recruiting
NCT04381936^(^^33^^)^	II-III	Hospitalized patients at high-risk of severe disease	LPV/Rit*versus* corticosteroid *versus* HyChl *versus* Azi *versus* Tmab associated with ST *versus* CP associated with ST	275mL±75mL on days 1 and 2	all	3/19/20	12/1/31	4.2	Recruiting

Passive immunotherapy occurs through the infusion of plasma from convalescent individuals, hence the use of the term convalescent plasma which can also be called hyperimmune plasma or ABO-compatible convalescent plasma.

CP: convalescent plasma; LPV: lopinavir; Rit: ritonavir; HydChl: hydroxychloroquine; IFN-β1a: interferon-β1a; Anak: anakinra; Tmab: tocilizumab; Smab: sarilumab; Vit C: vitamin C; ST: standard therapy; ICU: intensive care unit; SP: standard plasma; IgG: immunoglobulin G; NR: not report; HIg: human immunoglobulin; FFP: fresh frozen plasma; FP24: plasma frozen within 24 hours after phlebotomy; HT-CP: high-titer convalescent plasma; IV: intravenous; Azi: azithromycin.

### Convalescent plasma intervention

The CP intervention applied in all CTP was for inpatients with different degrees of disease impairment, especially for severe cases admitted to the ICU (58.3%) with or without invasive mechanical ventilation, and all patients received the ST for COVID-19. The intervention arms applied in these inpatients normally compared the CTP of plasma therapy plus ST *versus* only ST associated or not with some selected drugs, such as corticosteroid, antibiotics, antimalarials (hydroxychloroquine), anticoagulants, human immunoglobulin, antiviral drugs, among others.^(^^10^^,^^22^^,^^23^^,^^29^^,^^31^^,^^33^^)^ The CP therapy was applied mainly as single dose, with different volume of transfusion (45.8%), and the volume more frequently used was 500mL, in 20.8%,^(^^13^^,^^16^^,^^18^^,^^39^^)^ following by doses of 200 to 250mL in 8.3%,^(^^12^^,^^21^^)^ 400mL in 8.3%,^(^^19^^,^^39^^)^ 300mL in 4.2%,^(^^24^^)^ and 600mL in 4.2%.^(^^23^^)^ In the cases using more than one dose, the volume was two doses of 250mL,^(^^13^^,^^16^^,^^17^^,^^20^^,^^29^^,^^31^^,^^33^^)^ used in most of CTP, following by 2x200mL^(^^19^^,^^25^^,^^28^^,^^29^^,^^33^^)^ and 2x300mL.^(^^23^^,^^33^^)^ Plasma doses were derived from a single donor,^(^^13^^,^^16^^,^^20^^,^^23^^)^ or until two different donors in some CTP.^(^^13^^,^^16^^)^ Almost all CTP test the intervention in individuals aged over 18 years, with exception of two CTP (Table 1).

**Figure 2:**
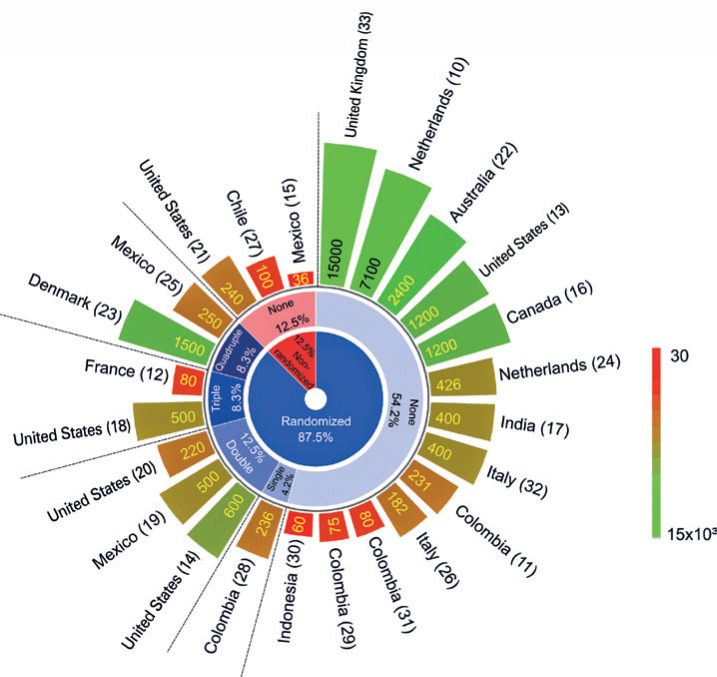
Study design of clinical trials of passive immunization against COVID-19 (plasma therapy), distributed inside out by the different types of allocation (randomized or not), masking (none, single, double, triple, and quadruple blinding), estimated enrollment (varied from 36 to 15,000 individuals), and study countries. The color scale bar represents the number of volunteers estimated in each protocol

Regarding study design characteristics of these CTP, figure 2 shows the intervention was mostly (87.5%) randomized and few CTP used some type of masking (33.3%), such as: 4.2% single blinding (participant), 12.5% double-blinding (participant and outcome evaluator), 8.3% triple-blinding (participant, care provider and outcome evaluator), and 8.3% quadruple-blinding (participant, care provider, investigator and outcome evaluator). Although 12.5% of CTP have not adopted any technique used to minimize the bias in allocations and blinding, keeping it open-label. The estimated enrollment of clinical trial in phase IV is 7,100 individuals,^(^^10^^)^ in phase III from 36 to 2,400 individuals,^(^^11^^–^^23^^)^ and phase II-III from 60 to 15,000.^(^^24^^–^^33^^)^ The number of volunteers estimated in each protocol was represented by the color scale bar in figure 2.

### Global research network in clinical trial protocol

**Figure 3:**
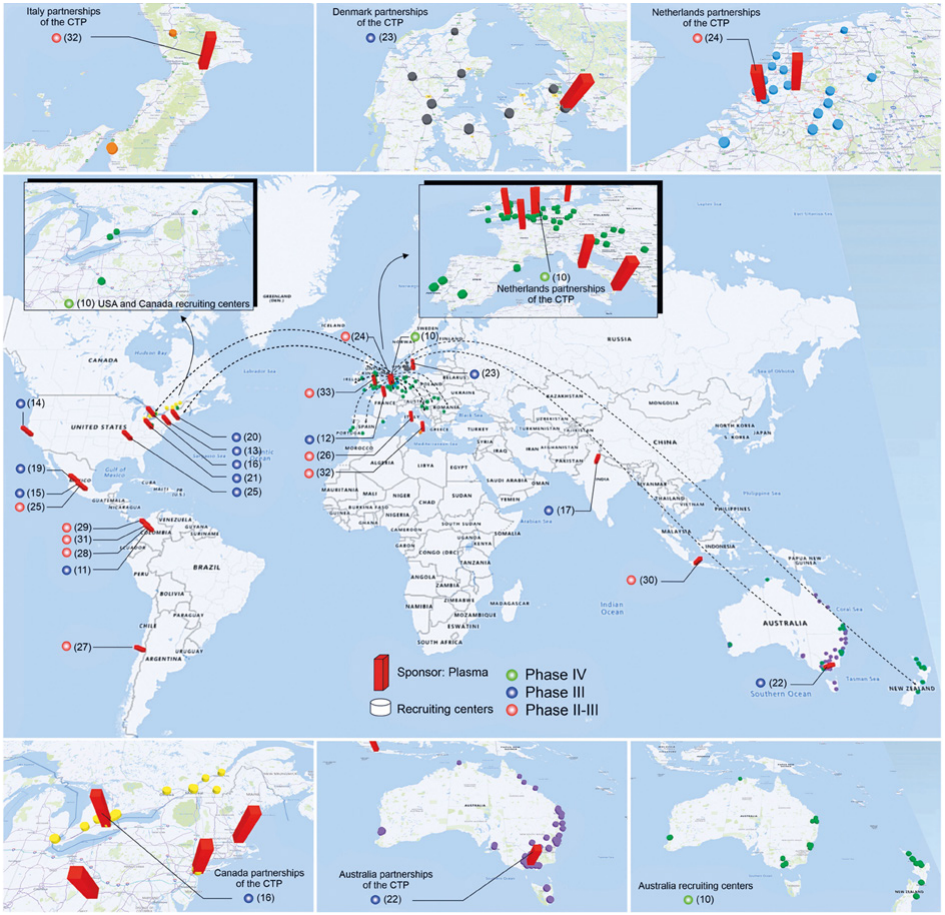
The global distribution of clinical trials by phase (circles) and the centers carrying out research on COVID-19 plasma therapy (red bar) and their recruitment centers (cylinders). The main centers of each continent are highlighted in the enlarged image around the central map. Phase IV is indicated by green circle; phase III by blue circle, and phases II-III by red circle. The intercontinental collaborations of the clinical trial protocols at phase IV are represented by the dashed black lines on the world map

Among the CP multicenter CTP,^(^^10^^,^^12^^,^^13^^,^^16^^,^^17^^,^^22^^–^^24^^,^^26^^,^^30^^,^^32^^)^ the CTP phase IV^(^^10^^)^ is the only one with intercontinental collaborations represented by the dashed black lines on the world map (Figure 3), with 87 recruitment centers (green cylinders) distributed in North America, Europe, and Oceania. The CTP phase III also have collaborations among countries and involve 48 recruitment centers (yellow cylinders) in the USA and Canada.^(^^16^^)^ The other multicenter CTP involve a varied number of recruitment centers within the same country, such as: Australia with 79 centers (purple cylinders),^(^^22^^)^ the Netherlands with 18 centers (blue cylinders),^(^^24^^)^ Denmark with 12 centers (dark gray cylinders),^(^^23^^)^ Italy with six centers (orange cylinders),^(^^32^^)^ highlighted in figure 3, with the enlarged image for better visualization of the collaboration centers, and others with approximately three centers.^(^^12^^,^^17^^,^^26^^,^^30^^)^ The single center CTP concentrate mainly in North America^(^^15^^,^^18^^–^^21^^)^ and South America.^(^^11^^,^^27^^–^^29^^,^^31^^)^

### Plasma therapy outcome in COVID-19 patients

The three studies that evaluate the efficacy of plasma therapy also prioritize the evaluation of COVID-19 in severely ill patients or those in life-threatening situation, such as the advanced clinical trials mentioned above. The CP therapy intervention varied a lot among studies, without a consensus about the best CP pattern of application. Regarding the results, the studies showed a reduction by 53% in disease severity (not needing intensive care),^(^^38^^)^ 26% in length of hospital stay,^(^^36^^)^ and from 35% to 50% in mortality rate,^(^^36^^–^^38^^)^ reporting adverse effects in less than 4% of patients after treatment with CP in different doses and volumes associated with standard therapy for COVID-19,^(^^36^^–^^38^^)^ as shown in table 2.

**Table 2:** Studies that evaluation the efficacy of plasma therapy in COVID-19 patients

References	n/CP sample	Antibody titer	CP dose (mL)	Viral charge (x10 ^/dL)	Previous treatments	Hospitalization (variation)	Adverse events (n)	Mortality rate reduction
Abolghasemi et al.^(^^36^^)^	189/115 CP *versus* 74 ST	Plasma antibody titer cut off index >1.1	2x500mL	NI	ST + antiviral (LPV/Rit), HydChl and anti-inflammatory agent	9.54 days CP *versus* 12.88 days ST CP reduced length of stay by ~26%	~1% CP Transient mild fever and chills following infusion of the plasma (n=1)	14.8% CP *versus* 24.3% ST ~40%
Li et al.^(^^37^^)^	103/52 CP (23 severe + 29 life-threatening) *versus* 51 ST (22 severe + 29 life-threatening)	1:350 (6 donors)	4-13mL/kg	Reduction in severe patients: 44.7% (24 hours), 68.1% (48 hours) and 87.2% (72 hours) virus-free Life-threatening patients: 53.8% (24 hours); 73.1 (48 hours) and 84.6% (72 hours) virus-free	Antiviral (41/46; 89.1%); antibacterial (38/46; 82.6%); Chinese herbal medicine (26/46; 56.5%); steroids (21/46 45.7%); antifungal (15/46; 32.6%); HIg (13/46; 28.3%); interferon (12/46; 26.1%)	Severe: 32.00 (26.00-40.00); life-threatening: indeterminate (46.00-indeterminate)	~4% CP Chills and rashes within 2 hours (n=1); shortness of breath, cyanosis and severe dyspnea within 6 hours (n=1)	15.7% CP *versus* 24.0% ST ~35%
Xia et al.^(^^38^^)^	1,568/138	Not significantly higher in rapid responders than in moderate responders	200-1,200mL	20/25 (80%) became virus-free after 14 days	NI	2.4% CP *versus* 5.1% ST CP reduced admitted to the ICU by ~53%	~2% CP Pruritus or erythema during transfusion (n=3)	2.2% CP *versus* 4.1% ST ~50%

CP: convalescent plasma; ST: standard treatment; NI: not informed; LPV: lopinavir; Rit: ritonavir; HydChl: hydroxychloroquine; HIg: human immunoglobulin; ICU: intensive care unit.

## DISCUSSION

In the absence of effective treatment for patients with COVID-19, many studies have sought alternatives to treat patients and enhance patient’s immune defense, such as the use of CP therapy. The recovery trial^(^^40^^)^ provides evidence to support some treatments (for instance, dexamethasone) and improve immunity in critical condition cases, and this trial uses CP therapy as one therapeutic arm. However, many aspects of this therapy are still being explored, such as a timeout/collection interval for COVID-19, or immunoglobulin G/immunoglobulin M (IgG/IgM) titers from donors, the therapy clinical improvement and efficacy in critical or non-critical patients and adverse effects. Among 170 CTP identified, only 24 CTP were in advanced phase (III/IV) with 33,000 individuals, concentrated in the regions of America, showing the pivotal questions on efficient use of the CP still uncertain or fragile to justify an increased use in critical or non-critical hospital care. In addition, no country, including the United States, has licensed CP as a treatment for COVID-19. The Food and Drug Administration (FDA) judged eligible for wider use under an emergency use authorization,^(^^41^^)^ although other countries have granted approval for use on an individual patient basis.

One of the first CP clinical trials that analyzed 103 patients with severe and life-threatening COVID-19 (median age 70 years),^(^^37^^)^ showed no statistically significance in clinical improvement after 28 days or reduced mortality. However, there was evidence of notable therapeutic effects and possible antiviral activity in group of 60 to 80-year-old patients, at the final stage of the disease course, after 14 days of symptoms, using only units with a very high antibody titers (IgG over 1:50) specific for spike (S)- and receptor-binding domain (RBD). Another study on CP therapy in severe COVID-19 patients^(^^42^^)^ showed significant improvement of clinical symptoms, with an increase in oxyhemoglobin saturation after the third day of transfusion, reduction of pulmonary lesions, amelioration of routine laboratory criteria, and pulmonary function accompanied by rapid neutralization of viremia, using 200mL of CP derived from recently recovered donors with the neutralizing antibody titers between 1:160-640, approximately 16.5 days after onset of symptoms, associated to standard care and antiviral agents. Only three CTP^(^^14^^,^^19^^,^^28^^)^ mentioned the antibody titers used in the CP therapy (over 1:160), and the studies by Abolghasemi et al.,^(^^36^^)^ and Li et al.,^(^^37^^)^ also reported the use of antibody titers above 1.1 in CP therapy associated with clinical improvement and reduction in mortality.

To reduce the variability in therapeutic response of patients, the WHO recommends some care and standardization in the selection of CP donors.^(^^43^^)^ Eligibility criterion in relation to donor age does not vary widely: 18-67 years.^(^^36^^,^^44^^)^ The donors were patients who recovered from COVID-19 and showed no detection of SARS-CoV-2 by real-time Quantitative polymerase chain reaction (qRT-PCR) or any related symptoms after a period that varied among studies. In one study, donors could have recovered after one week, and the short recovery period might have contributed to the death of 5 out of 6 patients.^(^^45^^)^ Longer recovery period allowed reports of therapeutic efficacy. This period could be 10 days, with collection performed twice, with a difference of 24 hours,^(^^46^^)^ at least 14 days,^(^^36^^,^^47^^)^ and more than two weeks.^(^^36^^,^^42^^,^^44^^)^ In some cases, qRT-PCR from nasopharyngeal swabs must be tested negative twice, and an interval of 24 hours between tests.^(^^36^^,^^42^^)^

Regarding the quantification of antibodies, S-RBD-specific IgG titers vary from donor to donor. One study demonstrated that ten out of 25 collected plasma displayed the titer of 1:450, 6/25 1:350, while in the others vary from 1:1 to 1:150.^(^^44^^)^ Most of studies employed a CP volume of roughly 500mL, in a single dose or divided into two doses, derived from a single donor,^(12,16,20,23)^ or two different donors.^(^^12^^,^^16^^)^ Therefore, this lack of standardization regarding donor selection, quality control of the CP, and recipient patients could explain the varied therapeutic effects.

Some possible adverse effects with the use of CP can be avoided, such as CP free of antigens, which could cause transfusion-related acute lung injury (TRALI), such as human leukocyte antigens that protect the embryo.^(^^36^^)^ In a multicenter clinical trial, the use of CP was not allowed in pregnant women, aiming to prevent TRALI.^(^^36^^)^

The severity of patient’s disease transfused with CP varied from mild, moderate, severe to critical. A CTP with severe patients divided the study groups in severe acute respiratory illness and severe community acquired pneumonia.^(^^10^^)^ Another CTP compared the effects of the treatment in oncological and non-oncological COVID-19 patients.^(^^27^^)^ Admission in the intensive care unit is related in some CTP,^(^^11^^,^^18^^,^^28^^,^^30^^)^ although it is presumed that it is applied to all severe patients. In some CTP, the CP treatment was compared to other treatments, such as corticoids, antibiotics, monoclonal antibodies, and anti-viral drugs.^(^^10^^,^^22^^,^^23^^,^^33^^)^

Some published results allowed evaluation of different parameters concerning the efficacy of treatment. In a study in which CP was applied, 6 out of 17 patients required mechanical ventilation, mainly elderly patients.^(^^47^^)^ In a multicenter study, the mortality rate was 14.8% of the patients (n=115).^(^^36^^)^ Similar results were found in another multicenter study (15.7%).^(^^36^^)^ Another investigation reported a mortality rate of only 2.2%.^(^^38^^)^ A study employed this therapy in patients with hypertension, diabetes or cardiovascular disease, but it was not clear the effect of these comorbidities in the CP treatment effect.^(^^36^^)^

The transfusion of CP therapy for COVID-19 must follow some pre-established conditions, such as availability of a population of donors who have recovered from the disease and can donate convalescent serum; blood banks to process serum donations; availability of assays, including serological tests, to detect SARS-CoV-2 in serum and virological assays to measure viral neutralization; laboratory support for virology to carry out these tests; and standardization of phase and condition of COVID-19 patient.^(^^48^^)^

The main limitations of the multicenter studies were the reduced number of patients in the control groups compared to the treatment group, usually due to lack of blood group CP match, and concomitant or previous use of another treatment.^(^^36^^)^ Another limitation is the lack of standard protocols and training for the study staff, as well as diversity in patients monitoring.^(^^49^^)^ In turn, the main limitation of our study is the impossibility of carrying out a meta-analysis, due to the lack of a robust number of studies reporting conclusive therapeutic effects of this modality, such as decrease in SARS-CoV-2 titers. However, few articles published on multicenter studies demonstrated that CP could be a promising therapeutic modality.

## CONCLUSION

Currently, there are no reliable therapeutic options for critically-ill COVID-19 patients. Based on the few consolidated multicenter clinical data results available, we concluded the convalescent plasma therapy studies provided relevant results in severe/critical cases of COVID-19 patients, reducing length of hospital stay, disease severity, and mortality, with low frequency of adverse events in a considerable number of patients. However, it is not possible to state, in a conclusive fashion, about the real relevance of this treatment, considering the lack of data that enable a robust statistics analysis, such as a meta-analysis.
