# Supplementary material for: Convalescent plasma therapy in COVID-19 critically ill patients during advanced phases of clinical trials and their preliminary results
Source: Einstein (Sao Paulo). 2021 Apr 9;19:eRW6186. doi: 10.31744/einstein_journal/2021RW6186 (PMC8034864; doi:10.31744/einstein_journal/2021RW6186)
Supplement: Translation

# Translation: Terapia com plasma convalescente em pacientes graves com COVID-19 nas fases avançadas dos ensaios clínicos e seus resultados preliminares

**Authors:** Fernando Anselmo de Oliveira, Mariana Penteado Nucci, Gabriel Nery de Albuquerque Rego, Arielly da Hora Alves, Luciana Cavalheiro Marti, Leopoldo Penteado Nucci, Javier Bustamante Mamani, Lionel Fernel Gamarra

**Affiliations:** 1 São PauloSP Brasil Hospital Israelita Albert Einstein, São Paulo, SP, Brasil.; 2 São PauloSP Brasil Hospital das Clínicas, Faculdade de Medicina, Universidade de São Paulo, São Paulo, SP, Brasil.; 3 GamaDF Brasil Centro Universitário do Planalto Central Apparecido dos Santos, Gama, DF, Brasil.

**Keywords:** COVID-19, Infecções por coronavírus, SARS-CoV-2, Betacoronavírus, Imunização, Imunização passiva, Plasma, Plasma convalescente

## Abstract

O objetivo deste estudo foi destacar o esforço científico global para combater o SARS-CoV-2 abordando os resultados preliminares da imunização passiva por plasma convalescente. Foi realizada uma busca nas principais bases de dados dos protocolos de ensaios clínicos intervencionistas sobre transfusão de plasma convalescente em pacientes com COVID-19, bem como artigos publicados (n≥25), utilizando a seguinte estratégia de busca: [(COVID-19 *OR* SARS-CoV-2 *OR* nCoV-2019) *AND* (*Convalescent plasma OR Plasma exchange*) *AND* (*Treatment OR Therapy*)]. Um total de 24 protocolos de ensaios clínicos intervencionistas (avançados nas fases II-III, III e IV) foi incluído nesta revisão, assim como três estudos que tiveram resultados suficientes para avaliar a eficácia da terapia com plasma convalescente para pacientes com COVID-19. Todos os protocolos de ensaios clínicos intervencionistas aplicaram cerca de 500mL de plasma convalescente (de uma ou mais doações) em pacientes hospitalizados, principalmente naqueles com grau grave de doença associada à terapia-padrão para COVID-19 em comparação com placebo ou terapia-padrão mais medicamentos específicos. A maioria dos protocolos de ensaios clínicos intervencionistas é multicêntrica, e os estudos de fase IV estão recrutando em centros intercontinentais da América do Norte, Oceania e Europa, mas a maior parte dos centros de recrutamento está dentro de seu próprio país. Os três estudos publicados relataram abordagem semelhante de intervenção para plasma convalescente com redução do tempo de internação, mortalidade e menos de 4% de eventos adversos, principalmente para o tratamento de casos críticos com risco de vida. Todos os ensaios clínicos avançados focaram na terapia com plasma convalescente em pacientes com COVID-19 hospitalizados em condições graves, e os resultados preliminares fornecem fortes evidências para a terapia para esses pacientes com COVID-19.

## INTRODUÇÃO

No final de 2019, surgiu o coronavírus da síndrome respiratória aguda grave 2 (SARS-CoV-2), resultando na doença pelo coronavírus 2019 (COVID-19), que causou uma emergência sanitária sem precedentes, sendo declarada pandemia pela Organização Mundial de Saúde (OMS) em 11 de março de 2020.^(^^1^^)^ Até setembro de 2020, essa pandemia afetara cerca de 28 milhões pessoas, com cerca de 920 mil mortes em todo o mundo.^(^^2^^)^ No Brasil, até agora, já ocorreram mais de 5 milhões de casos com mais de 130 mil mortes.^(^^3^^)^

Novas abordagens para o desenvolvimento de transferência de imunidade foram rapidamente implementadas em estudos pré-clínicos, e, atualmente, surgem resultados interessantes de ensaios clínicos, com o objetivo de melhorar os sintomas da COVID-19, uma doença heterogênea.^(^^2^^)^ Ao mesmo tempo, diversas vacinas estão sendo desenvolvidas e testadas para a prevenção efetiva da doença.^(^^4^^)^

Antes da SARS-CoV-2, já tinha sido investigado o uso de plasma convalescente (PC), com desfechos positivos, em surtos de outras infecções virais,^(^^5^^)^ como a pandemia de gripe A (H1N1), em 2009,^(^^6^^)^ e a gripe aviária A (H5N1),^(^^7^^)^ entre outras. Além disso, há estudos que demonstram que os anticorpos do PC podem limitar a proliferação do vírus durante a infecção e promover a eliminação viral, favorecendo uma recuperação rápida da doença.^(^^8^^)^

Revisão recente sobre a terapia com plasma em pacientes com COVID-19 relatou baixa frequência de eventos adversos graves e melhora nos sintomas clínicos em alguns participantes após a terapia com plasma, mas os autores julgaram existir risco de viés de relatório.^(^^9^^)^

Os títulos altos ou baixos de anticorpos neutralizantes contra a COVID-19 podem ser manejados para reduzir os sintomas e a mortalidade do paciente. Existem 24 ensaios clínicos avançados nas fases II-III, III e IV relatados em vários países usando PC para tratar esses pacientes e responder a essa pergunta.^(^^10^^–^^33^^)^ Embora a imunização passiva seja utilizada há mais de um século no tratamento de doenças infecciosas, resultados recentes apresentam desafios na definição do melhor momento de extração do plasma e na escolha do doador, além do custo de todo esse procedimento. Essa falta de informação potencializa os movimentos de grupos antivacina e antiplasma.^(^^34^^)^

## OBJETIVO

Destacar o esforço científico global na luta contra o SARS-CoV-2, abordando a imunização passiva por plasma convalescente e seus resultados preliminares.

## MÉTODOS

Foi realizada uma pesquisa até 14 de setembro de 2020 em ClinicalTrials.gov (https://clinicaltrials.gov/), *Chinese Clinical Trial Registry* (http://www.chictr.org.cn/abouten.aspx) e *EU Clinical Trials Register* (https://www.clinicaltrialsregister.eu/) para ensaios clínicos de intervenção sobre transfusão de PC, em pacientes com COVID-19, usando a seguinte estratégia de pesquisa: [(COVID-19 *OR* SARS-CoV-2 *OR* nCoV-2019) *AND* (*Convalescent Plasma OR Plasma Exchange*) *AND* (*Treatment OR Therapy*)]. Em seguida, a mesma estratégia foi usada para pesquisar estudos nas bases de dados PubMed^®^ e Scopus sobre a eficácia da terapia com PC para tratar pacientes com COVID-19.

### Critérios de inclusão e exclusão

Esta revisão incluiu protocolos de ensaios clínicos (PEC) nas fases III e IV que abordassem o desenvolvimento de terapias baseadas em PC para tratamento de pacientes com COVID-19 por imunização passiva e estudos que mostrassem a eficácia da terapia com PC aplicada em mais de 25 pacientes com COVID-19. Os motivos para exclusão de estudos foram os seguintes: PEC de estudos observacionais, PEC envolvendo vacinas e PEC cancelados ou não aprovados até a data da pesquisa nas bases de dados.

### Elegibilidade dos estudos, extração de dados, coleta de dados e avaliação de risco de viés

A elegibilidade dos estudos seguiu as diretrizes *Preferred. Reporting Items for Systematic Reviews and Meta-Analyses* (PRISMA).^(^^35^^)^

### Análise dos dados

Todos os resultados foram descritos e apresentados por meio de distribuição percentual para todas as variáveis analisadas nas tabelas.

## RESULTADOS

### Seleção de estudos

Após a aplicação das estratégias de busca nas bases de dados, 170 PEC foram identificados (153 protocolos no ClinicalTrials.gov, 14 no *Chinese Clinical Trial Registry* e três no *EU Clinical Trials Register*). A estratégia de busca utilizou o PRISMA.^(^^35^^)^

Com base nos critérios de inclusão e exclusão estabelecidos, dentre os 170 protocolos identificados, 146 ensaios clínicos foram excluídos após a triagem (130 protocolos eram fases I e II, e 16 eram observacionais), restando 24 protocolos selecionados dessas bases de dados. No total, 24 PEC foram incluídos neste trabalho sobre imunização passiva para COVID-19 por meio de terapia com PC.^(^^10^^–^^13^^,^^15^^–^^33^^)^

Dentre os estudos selecionados publicados nas bases de dados citadas, apenas três estudos apresentaram dados suficientes que permitiam uma análise estatística dos desfechos, para avaliar a eficácia da terapia com PC devido ao número de pacientes com COVID-19 (n≥25).^(^^36^^–^^38^^)^

### Visão geral dos protocolos de ensaios clínicos sobre imunização passiva para COVID-19

Dos 24 ensaios clínicos mais avançados sobre terapia com PC para pacientes hospitalizados com COVID-19, apenas um (4,2%) estava na fase IV, com taxa de progresso do estudo (TPE) de 58%, recrutando indivíduos em diferentes países pelo patrocinador holandês (barra verde da figura 1).^(^^10^^)^ Nos estudos de fase III (54,2%),^(^^11^^–^^13^^,^^15^^–^^19^^)^ quase metade dos PEC tinha TPE de mais de 40%, principalmente no México^(^^15^^,^^19^^)^ e nos Estados Unidos (barras azuis da figura 1),^(^^18^^,^^21^^)^ e, nos estudos de fase II-III (41,7%),^(^^24^^–^^33^^)^ um PEC tinha TPE de 100% na Holanda,^(^^24^^)^ e quase metade dos PEC tinha TPE de mais de 40% (barras vermelhas da figura 1), conforme mostrado na figura 1 e na tabela 1.

**Figura 1:** Análise do percentual da taxa de progresso do estudo de protocolos de ensaios clínicos sobre terapia de plasma convalescente para pacientes com COVID-19 hospitalizados distribuídos por suas fases (barra verde: fase IV; barras azuis: fase III; e barras vermelhas: fase II-III), com o correspondente país patrocinador

**Tabela 1:** Desenho do estudo, braços, intervenções e progresso do tempo de estudo

Número de identidade	Fase	Características do paciente	Intervenção por braço	Dose PC (dia)	Faixa etária (anos)	Data de início	Data de conclusão	Progresso (%)	Situação de recrutamento
NCT02735707^(^^10^^)^	IV	Doença respiratória aguda grave e pneumonia adquirida na comunidade grave	Corticosteroides *versus* antibióticos *versus* macrolídeo *versus* antiviral para gripe *versus* LPV/Rit *versus* HCQ *versus* IFN-β1a *versus* Anak *versus* Tmab *versus* Smab *versus* Vit C *versus* heparina *versus* sinvastatina *versus* PC *versus* TP	1 ou 2 unidades em 48 horas	>18	11/4/2016	1/12/2023	58	Recrutamento
NCT04391101^(^^11^^)^	III	Pacientes graves tratados na UTI	PC associado a TP *versus* TP	400-500mL	>18	1/6/2020	1/12/2021	19,2	Ainda sem recrutamento
NCT04372979^(^^12^^)^	III	Atendimento precoce de pacientes hospitalizados	PC associado a TP *versus* PP associado a TP	200-230mL	18-80	1/5/2020	1/5/2021	37,3	Ainda sem recrutamento
NCT04418518^(^^13^^)^	III	Atendimento precoce a pacientes hospitalizados	PC associado a TP *versus* TP	500mL de doador único ou 2 unidades de 250mL de 1-2 doações	18-70	24/6/2020	1/12/2021	15,6	Recrutamento
NCT04355767^(^^14^^)^	III	Pacientes graves/críticos hospitalizados	PC (títulos de anticorpos ≥1:160) *versus* placebo	PC: 1 unidade *versus* placebo: solução fisiológica com multivitamínico	>18	11/8/2020	1/12/2022	4,0	Ainda sem recrutamento
NCT04432103^(^^15^^)^	III	Pacientes graves/críticos hospitalizados	PC de IgG (pacientes graves *versus* pacientes críticos) associado a TP	NR	>19	19/6/2020	30/9/2020	84,5	Ainda sem recrutamento
NCT04348656^(^^16^^)^	III	Atendimento precoce a pacientes hospitalizados	PC *versus* TP	500mL de doador único ou 2 unidades de 250mL de 1-2 doações	>20	14/5/2020	31/12/2020	53,2	Recrutando
NCT04425915^(^^17^^)^	III	Pacientes graves/críticos hospitalizados	PC associado a TP *versus* TP	2 doses de 250mL em dias consecutivos iniciadas no dia 3 do início dos sintomas	>21	14/6/2020	30/5/2021	26,3	Recrutando
NCT04362176^(^^18^^)^	III	Pacientes hospitalizados ou em UTI	Patógeno reduzido PC *versus* placebo	PC: 500mL em 12 horas (dia 0) *versus* placebo: 250mL de Ringer lactato associado a multivitaminas (dia 1)	>22	24/4/2020	1/4/2021	41,8	Recrutando
NCT04381858^(^^19^^)^	III	Insuficiência respiratória grave com ventilação mecânica invasiva	PC (títulos de anticorpos > 1:164) *versus* HIg	PC: 400mL (2 unidades) *versus* HIg 0,3g/kg/dia (5 doses)	16-18	6/5/2020	30/9/2020	89,1	Recrutando
NCT04361253^(^^20^^)^	III	Pacientes hospitalizados	PC (título alto) *versus* PP (PFC ou PC 24)	PC-AT 2 doses de 250mL de doador único em 24 horas; PFC: 2 unidades de 200-275mL	>1	30/4/2020	1/12/2021	23,6	Recrutando
NCT04376034^(^^21^^)^	III	Gravidade leve, moderada e grave/crítica	PC associado a TP *versus* TP	Adultos: 200 a 250mL; crianças: 10mL/kg; 2 unidades de pacientes graves ou em condição crítica	>30 dias	16/4/2020	30/3/2021	43,4	Recrutando
NCT04483960^(^^22^^)^	III	Sem pacientes graves	LPV/Rit *versus* HCQ *versus* PC	1 unidade no dia 1 e no dia 2	>18	21/7/2020	12/6/2022	8,0	Recrutando
NCT04345289^(^^23^^)^	III	Pacientes hospitalizados com doença leve, moderada e grave/crítica	PC associado a TP (Smab *versus* baricitinibe *versus* HCQ) *versus* TP associado a placebo injetável	PC: (2 vezes de 300mL) e dose única de 600mL; placebo: (2 vezes de 300mL) e dose única de 600mL de solução fisiológica IV por placebo: 3 vezes ao dia (7 dias)	>18	1/5/2020	15/6/2021	33,2	Recrutando
NCT04342182^(^^24^^)^	II-III	Pacientes hospitalizados	PC associado a TP *versus* TP	300mL (de acordo com o protocolo Erasmus MC KIS)	>18	8/4/2020	1/7/2020	100	Recrutando
NCT04388410^(^^25^^)^	II-III	Pacientes hospitalizados com doença grave ou risco de doença grave	PC *versus* placebo	PC: 2 unidades de 200mL dentro de 24-72 horas; placebo: 200mL de soro fisiológico	>18	25/8/2020	31/12/2020	15,6	Ainda sem recrutamento
NCT04374526^(^^26^^)^	II-III	Pacientes hospitalizados	PC associado a TP *versus* TP	200mL/dia por 3 dias	>65	27/5/2020	30/6/2021	27,6	Recrutando
NCT04384588^(^^27^^)^	II-III	Pacientes oncológicos e não oncológicos com doença grave	PC associado a TP *versus* TP	1 ou mais unidades	>15	7/4/2020	6/4/2021	44,0	Recrutando
NCT04425837^(^^28^^)^	II-III	Pacientes hospitalizados com alto risco de doença grave ou em UTI	PC (títulos de anticorpos ≥1:160) associado a TP *versus* TP	2 doses de 200mL por dia	>18	1/7/2020	1/2/2021	34,9	Ainda sem recrutamento
NCT04395170^(^^29^^)^	II-III	Atendimento precoce a pacientes hospitalizados	PC associado a TP *versus* anti-COVID-19 HIg *versus* TP	PC: 200-250mL (dias 1 e 3); Solução IgG a 10%: 50mL (paciente ≥50kg); 1mL/kg (paciente <50Kg), nos dias 1 e 3	>18	1/9/2020	1/6/2021	4,8	Ainda sem recrutamento
NCT04380935^(^^30^^)^	II-III	Pacientes hospitalizados na UTI (usando ventilação mecânica)	PC associado a TP *versus* TP	NR	>18	18/5/2020	31/10/2020	71,7	Ainda sem recrutamento
NCT04332835^(^^31^^)^	II-III	Gravidade moderada e grave/crítica	PC associado a TP *versus* HCQ associado a TP	250mL nos dias 1 e 2	18-60	8/8/2020	31/10/2020	44,0	Ainda sem recrutamento
NCT04385043^(^^32^^)^	II-III	Pacientes graves/críticos hospitalizados	PC associado a TP *versus* TP	NR	18-60	1/5/2020	15/5/2021	35,9	Recrutando
NCT04381936^(^^33^^)^	II-III	Pacientes hospitalizados com alto risco de doença grave	LPV/Rit *versus* corticosteroide *versus* HCQ *versus* Azi *versus* Tmab associado a TP *versus* PC associado a TP	275mL±75mL nos dias 1 e 2	Todos	19/3/2020	1/12/2031	4,2	Recrutamento

A imunoterapia passiva ocorre por meio da infusão de plasma de indivíduos convalescentes, por isto o uso do termo plasma convalescente que também pode ser denominado plasma hiperimune ou plasma convalescente compatível com ABO (tipo sanguíneo).

PC: plasma convalescente; LPV: lopinavir; Rit: ritonavir; HCQ: hidroxicloroquina; IFN-β1a: interferon beta-1a; Anak: anakinra; Tmab: tocilizumabe; Smab: sarilumabe; Vit C: vitamina C; TP: tratamento padrão; UTI: unidade de terapia intensiva; PP: plasma padrão; IgG: imunoglobulina G; NR: não reportar; HIg: imunoglobulina humana; PFC: plasma fresco congelado; PC24: plasma congelado em 24 horas após a flebotomia; PC-AT plasma convalescente de alto título; IV: intravenoso; Azi: azitromicina.

### Intervenção com plasma convalescente

A intervenção com PC aplicada em todos os PEC foi para pacientes hospitalizados com diferentes graus de comprometimento da doença, principalmente para os casos graves internados em UTI (58,3%) com ou sem ventilação mecânica invasiva, e todos os pacientes receberam o TP para COVID-19. Os braços de intervenção aplicados nesses pacientes hospitalizados normalmente comparavam o PEC da terapia com plasma mais TP em relação a somente TP associado ou não a alguns medicamentos selecionados, como corticosteroides, antibióticos, antimaláricos (hidroxicloroquina), anticoagulantes, imunoglobulina humana, medicamentos antivirais, entre outros.^(^^10^^,^^22^^,^^23^^,^^29^^,^^31^^,^^33^^)^ A terapia com PC foi aplicada principalmente por meio de dose única com diferentes volumes de transfusão (45,8%). O volume mais utilizado foi de 500mL, em 20,8%,^(^^13^^,^^16^^,^^18^^,^^39^^)^ seguido de doses de 200 a 250mL em 8,3%,^(^^12^^,^^21^^)^ 400mL em 8,3%,^(^^19^^,^^39^^)^ 300mL em 4,2%,^(^^24^^)^ e 600mL em 4,2%.^(^^23^^)^ Nos casos com mais de uma dose, o volume foi de duas doses de 250mL,^(^^13^^,^^16^^,^^17^^,^^20^^,^^29^^,^^31^^,^^33^^)^ utilizadas na maioria dos PEC, seguidas de 2x200mL^(^^19^^,^^25^^,^^28^^,^^29^^,^^33^^)^ e 2x300mL.^(^^23^^,^^33^^)^ As doses de plasma foram derivadas de um único doador^(^^13^^,^^16^^,^^20^^,^^23^^)^ ou de até dois doadores diferentes em alguns PEC.^(^^13^^,^^16^^)^ Quase todos os PEC testaram a intervenção em indivíduos com mais de 18 anos, com exceção de dois PEC (Tabela 1).

Em relação às características do desenho do estudo desses PEC, a figura 2 mostra que a intervenção foi randomizada em sua maioria (87,5%), e poucos PEC utilizaram algum tipo de mascaramento (33,3%), como 4,2% com cegamento simples (participante), 12,5% com cegamento duplo (participante e avaliador dos desfechos), 8,3% com cegamento triplo (participante, prestador de cuidados e avaliador dos desfechos) e 8,3% com cegamento quádruplo (participante, prestador de cuidados, investigador e avaliador dos desfechos). No entanto, 12,5% dos PEC não adotaram nenhuma técnica utilizada para minimizar o viés nas alocações e no cegamento, mantendo-se *open-label*. A inscrição estimada em ensaios clínicos em fase IV é de 7.100 indivíduos,^(^^10^^)^ em fase III é de 36 a 2.400 indivíduos^(^^11^^–^^23^^)^ e, em fase II-III, de 60 a 15 mil indivíduos.^(^^24^^–^^33^^)^ O número estimado de voluntários em cada protocolo está representado pela barra de escala colorida na figura 2.

**Figura 2:** Desenhos de estudo de ensaios clínicos de imunização passiva contra a COVID-19 (terapia com plasma), distribuídos sistematicamente pelos diferentes tipos de alocação (randomizada ou não), mascaramento (nenhum, cegamento único, duplo, triplo e quádruplo), inscrição estimada (variada de 36 a 15 mil indivíduos) e países de estudo. A barra de escala colorida representa o número estimado de voluntários em cada protocolo

### Rede global de pesquisa em protocolos de ensaios clínicos

Entre os PEC multicêntricos sobre PC,^(^^10^^,^^12^^,^^13^^,^^16^^,^^17^^,^^22^^–^^24^^,^^26^^,^^30^^,^^32^^)^ o de fase IV^(^^10^^)^ é o único com colaborações intercontinentais representadas pelas linhas pretas tracejadas no mapa-múndi (Figura 3), com 87 centros de recrutamento (cilindros verdes) distribuídos na América do Norte, na Europa e na Oceania. Os PEC de fase III também têm colaboração entre países e envolvem 48 centros de recrutamento (cilindros amarelos) nos Estados Unidos e no Canadá.^(^^16^^)^ Os outros PEC multicêntricos envolvem um número variado de centros de recrutamento dentro do mesmo país, como Austrália com 79 centros (cilindros roxos),^(^^22^^)^ Holanda com 18 centros (cilindros azuis),^(^^24^^)^ Dinamarca com 12 centros (cilindros cinza escuro),^(^^23^^)^ Itália com seis centros (cilindros laranja),^(^^32^^)^ destacados na figura 3, com a imagem ampliada para melhor visualização do centros de colaboração e outros com cerca de três centros.^(^^12^^,^^17^^,^^26^^,^^30^^)^ Os PEC de centro único concentram-se principalmente na América do Norte^(^^15^^,^^18^^–^^21^^)^ e América do Sul.^(^^11^^,^^27^^–^^29^^,^^31^^)^

**Figura 3:** Distribuição global dos ensaios clínicos por fase (círculos) e os centros que desenvolvem pesquisas em terapia com plasma para COVID-19 (barra vermelha), além de seus centros de recrutamento (cilindros). Os principais centros de cada continente estão destacados na imagem ampliada em torno do mapa central. Fase IV está indicada por **círculo verde**; fase III, por círculo azul e fases II-III por círculo vermelho. As colaborações intercontinentais dos protocolos de ensaios clínicos de fase IV estão representadas pelas linhas pretas tracejadas no mapa mundial

### Desfecho da terapia com plasma em pacientes COVID-19

Os três estudos que avaliam a eficácia da terapia com plasma também priorizam a avaliação da COVID-19 em pacientes gravemente doentes ou com risco de vida, como os ensaios clínicos avançados mencionados. A intervenção da terapia com PC variou muito entre os estudos, sem um consenso sobre o melhor padrão de aplicação de PC. Em relação aos resultados, os estudos mostraram redução de 53% na gravidade da doença (dispensando terapia intensiva),^(^^38^^)^ de 26% no tempo de internação^(^^36^^)^ e de 35% a 50% na mortalidade,^(^^36^^–^^38^^)^ relatando efeitos adversos em menos de 4% dos pacientes após tratamento com PC em diferentes doses e volumes associados à terapia padrão para COVID-19,^(^^36^^–^^38^^)^ conforme mostrado na tabela 2.

**Tabela 2:** Estudos que avaliam a eficácia da terapia com plasma em pacientes com COVID-19

Referências	Amostra n/PC	Título de anticorpos	Dose PC (mL)	Carga viral (x10 ^/dL)	Tratamentos anteriores	Hospitalização (variação)	Eventos adversos (n)	Redução da taxa de mortalidade
Abolghasemi et al.^(^^36^^)^	189/115 PC *versus* 74 TP	Índice de corte de título de anticorpos plasmáticos >1,1	2×500mL	NI	TP + antiviral (LPV/Rit), HCQ e agente anti-inflamatório	9,54 dias PC *versus* 12,88 dias TP PC reduziu o tempo de hospitalização em aproximadamente 26%	Aproximadamente 1% PC Febre leve transitória e calafrios após infusão do plasma (n=1)	14,8% PC *versus* 24,3% TP Aproximadamente 40%
Li et al.^(^^37^^)^	103/52 PC (23 graves + 29 com risco de vida) *versus* 51 TP (22 graves + 29 com risco de vida)	1:350 (6 doadores)	4-13mL/kg	Redução em pacientes graves: 44,7% (24 horas), 68,1% (48 horas) e 87,2% (72 horas) livre de vírus Pacientes com risco de vida: 53,8% (24 horas); 73,1 (48 horas) e 84,6% (72 horas) livre de vírus	Antiviral (41/46; 89,1%); antibacteriano (38/46; 82,6%); fitoterapia chinesa (26/46; 56,5%); corticoides 21/46; 45,7%); antifúngico (15/46; 32,6%); HIg (13/46; 28,3%); Interferon (12/46; 26,1%)	Graves: 32,00 (26,00-40,00); com risco de vida: indeterminado (46,00 - indeterminado)	Aproximadamente 4% PC Calafrios e erupções cutâneas em 2 horas (n=1); falta de ar, cianose e dispneia intensa em 6 horas (n=1)	15,7% PC *versus* 24,0% TP Aproximadamente 35%
Xia et al.^(^^38^^)^	1.568/138	Não significativamente maior nos que responderam de forma rápida ao tratamento do que nos que responderam de forma moderada	200-1.200mL	20/25 (80%) ficaram livres de vírus após 14 dias	NI	2,4% PC *versus* 5,1% TP PC reduziu o número de internados na UTI em aproximadamente 53%	Aproximadamente 2% PC Prurido ou eritema durante a transfusão (n=3)	2,2% PC *versus* 4,1% TP Aproximadamente 50%

PC: plasma convalescente; TP: tratamento padrão; NI: não informado; LPV: lopinavir; Rit: ritonavir; HCQ: hidroxicloroquina; HIg: imunoglobulina humana; UTI: unidade de terapia intensiva.

## DISCUSSÃO

Na ausência de um tratamento efetivo para pacientes com COVID-19, muitos estudos têm buscado alternativas para tratar os pacientes e melhorar sua defesa imune, como é o caso do uso da terapia com PC. O ensaio de recuperação^(^^40^^)^ fornece evidências para apoiar algumas formas de tratamento (por exemplo, dexametasona) e de melhora da imunidade em pacientes em condições críticas, e esse ensaio utiliza a terapia com PC como braço terapêutico. No entanto, muitos aspectos dessa terapia ainda estão sendo explorados, como o intervalo de tempo limite e o momento ideal de coleta para a COVID-19, ou os títulos de imunoglobulina G/imunoglobulina M (IgG/IgM) dos doadores, a melhora clínica proporcionada pela terapia, sua eficácia em pacientes críticos ou não críticos e seus efeitos adversos. Dentre os 170 PEC identificados, apenas 24 PEC estavam em fase avançada (III/IV) com 33 mil indivíduos, concentrados nas regiões da América, mostrando as principais questões referentes à eficácia do uso do PC ainda incertas ou frágeis para justificar a ampliação de seu uso no atendimento hospitalar de pacientes críticos ou não críticos. Além disso, nenhum país, incluindo os Estados Unidos, licenciou o PC como tratamento para a COVID-19. *A Food and Drug Administration* (FDA) o considerou elegível para uso mais amplo sob uma autorização de uso de emergência,^(^^41^^)^ embora outros países tenham concedido aprovação para seu uso em alguns pacientes.

Um dos primeiros ensaios clínicos com PC que analisou 103 pacientes com COVID-19 grave e risco de vida (idade mediana de 70 anos)^(^^37^^)^ não mostrou significância estatística na melhora clínica após 28 dias ou redução da mortalidade. No entanto, houve evidências de efeitos terapêuticos notáveis e possível atividade antiviral em grupos de pacientes de 60 a 80 anos, no estágio final da evolução da doença, após 14 dias de sintomas, utilizando apenas unidades com títulos de anticorpos muito altos (IgG superior a 1:50) específicos para *spike* (S) e domínio de ligação ao receptor (RBD). Outro estudo sobre o tratamento com PC em pacientes com COVID-19 grave^(^^42^^)^ mostrou melhora significativa dos sintomas clínicos, com aumento da saturação da oxi-hemoglobina após o terceiro dia de transfusão, redução das lesões pulmonares, melhora dos critérios laboratoriais de rotina e da função pulmonar acompanhada por rápida neutralização da viremia, utilizando 200mL de PC proveniente de doadores recentemente recuperados com títulos de anticorpos neutralizantes entre 1:160-640, cerca de 16,5 dias após o início dos sintomas, associados ao tratamento padrão e agentes antivirais. Apenas três PEC^(^^14^^,^^19^^,^^28^^)^ mencionaram os títulos de anticorpos usados na terapia com PC (acima de 1:160), e os estudos de Abolghasemi et al.,^(^^36^^)^ e Li et al.,^(^^37^^)^ também relataram o uso de títulos de anticorpos acima de 1.1 na terapia com PC associados à melhora clínica e à redução da mortalidade.

Para reduzir a variabilidade na resposta terapêutica dos pacientes, a OMS recomenda alguns cuidados e padronização na seleção de doadores de PC.^(^^43^^)^ O critério de elegibilidade em relação à idade do doador não varia muito: 18 a 67 anos.^(^^36^^,^^44^^)^ Os doadores eram pacientes que se recuperaram de COVID-19 e não mostraram detecção de SARS-CoV-2 por reação em cadeia da polimerase quantitativa em tempo real (qRT-PCR) ou quaisquer sintomas relacionados após um período que variou entre os estudos. Em um estudo, os doadores podem ter se recuperado após 1 semana, e o curto período de recuperação talvez possa ter contribuído para a morte de cinco em seis pacientes.^(^^45^^)^ Recuperação mais longa permitiu relatos de eficácia terapêutica. Esse período pode ser de 10 dias, com a coleta realizada em duas vezes, com diferença de 24 horas,^(^^46^^)^ de pelo menos 14 dias^(^^36^^,^^47^^)^ e de mais de 2 semanas.^(^^36^^,^^42^^,^^44^^)^ Em alguns casos, o qRT-PCR dos *swabs* nasofaríngeos deve ser testado como negativo duas vezes, com intervalo de 24 horas entre os exames.^(^^36^^,^^42^^)^

Em relação à quantificação de anticorpos, os títulos de IgG específicos para S-RBD variam de doador para doador. Estudo demonstrou que dez em 25 amostras de plasma coletado exibiram o título de 1:450, 6/25 1:350, ao passo que, nos demais, variaram de 1:1 a 1:150.^(^^44^^)^ A maioria dos estudos utilizou um volume de PC em torno de 500mL, em dose única ou dividido em duas doses, proveniente de um único doador^(^^12^^,^^16^^,^^20^^,^^23^^)^ ou de dois doadores diferentes.^(^^12^^,^^16^^)^ Portanto, essa falta de padronização na seleção dos doadores, no controle de qualidade do PC e nos pacientes receptores poderia explicar os diversos efeitos terapêuticos.

Alguns possíveis efeitos adversos com o uso do PC podem ser evitados, como o uso de PC sem antígenos, que poderia causar lesão pulmonar aguda associada à transfusão (TRALI - *transfusion-related acute lung injury*), como os antígenos leucocitários humanos que protegem o embrião.^(^^36^^)^ Em ensaio clínico multicêntrico, não foi permitido o uso de PC em mulheres grávidas, com o objetivo de prevenir a ocorrência de TRALI.^(^^36^^)^

A gravidade da doença do paciente transfundido com PC variou de leve, moderada, grave a crítica. Um PEC com pacientes graves dividiu os grupos de estudo em pacientes com doença respiratória aguda grave e pneumonia grave adquirida na comunidade.^(^^10^^)^ Outro PEC comparou os efeitos do tratamento em pacientes com COVID-19 oncológicos e não oncológicos.^(^^27^^)^ A admissão na unidade de terapia intensiva está relatada em alguns PEC,^(^^11^^,^^18^^,^^28^^,^^30^^)^ embora se presuma que isso se aplique a todos os pacientes graves. Em alguns PEC, o tratamento com PC foi comparado a outros tratamentos, como corticoides, antibióticos, anticorpos monoclonais e medicamentos antivirais.^(^^10^^,^^22^^,^^23^^,^^33^^)^

Alguns resultados publicados permitiram a avaliação de diferentes parâmetros quanto à eficácia do tratamento. Em estudo em que foi aplicado o PC, seis de 17 pacientes necessitaram de ventilação mecânica - na maioria pacientes idosos.^(^^47^^)^ Em estudo multicêntrico, a taxa de mortalidade foi de 14,8% dos pacientes (n=115).^(^^36^^)^ Resultados semelhantes foram encontrados em outro estudo multicêntrico (15,7%).^(^^36^^)^ Em outro, a mortalidade foi de apenas 2,2%.^(^^38^^)^ Um estudo utilizou essa terapia em pacientes com hipertensão, diabetes ou doença cardiovascular, mas não ficou claro o efeito dessas comorbidades no resultado do tratamento com PC.^(^^36^^)^

A transfusão da terapia com PC para COVID-19 deve seguir algumas condições preestabelecidas, como disponibilidade de uma população de doadores que já se recuperou da doença e pode doar soro convalescente; bancos de sangue para processar as doações de soro; disponibilidade de testes, incluindo testes sorológicos, para detectar SARS-CoV-2 em exames séricos e virológicos para medir a neutralização viral; apoio laboratorial de virologia para a realização desses exames e padronização da fase e condição do paciente com COVID-19.^(^^48^^)^

As principais limitações dos estudos multicêntricos foram o número reduzido de pacientes nos grupos de controle em comparação ao grupo de tratamento, geralmente devido à falta de compatibilidade do grupo sanguíneo do PC e ao uso concomitante ou prévio de outro tratamento.^(^^36^^)^ Outra limitação é a falta de protocolos padronizados e treinamento para a equipe do estudo, além de diversidade no monitoramento dos pacientes.^(^^49^^)^ Por sua vez, a principal limitação do nosso estudo foi a impossibilidade de realizar uma metanálise, devido à falta de um número robusto de estudos que relatassem efeitos terapêuticos conclusivos dessa modalidade, como diminuição dos títulos de SARS-CoV-2. No entanto, alguns artigos publicados sobre estudos multicêntricos demonstraram que o PC pode ser uma modalidade terapêutica promissora.

## CONCLUSÃO

Atualmente, não há opções terapêuticas confiáveis para pacientes com COVID-19 em estado crítico. Com base nos poucos resultados de dados clínicos multicêntricos consolidados disponíveis, concluímos que os estudos de terapia com plasma convalescente forneceram resultados relevantes em casos graves/críticos de pacientes com COVID-19, reduzindo o tempo de internação, a gravidade da doença e a mortalidade, com baixa frequência de eventos adversos em um número considerável de pacientes. Porém, não é possível afirmar, de forma conclusiva, qual a real relevância desse tratamento, devido à falta de dados que permitam uma análise estatística robusta, como uma metanálise.
